# Cellular Senescence in Age-Related Diseases: Molecular Bases and Therapeutic Interventions

**DOI:** 10.3390/cells11132029

**Published:** 2022-06-26

**Authors:** Erica Buoso, Alessandro Attanzio, Fabrizio Biundo

**Affiliations:** 1 Department of Drug Sciences, University of Pavia, Via Taramelli 12/14, 27100 Pavia, Italy; erica.buoso@unipv.it; 2 Department of Pharmacology and Experimental Therapeutics, Boston University School of Medicine, Boston, MA 02118, USA; 3 Department of Biological, Chemical and Pharmaceutical Sciences and Technologies, University of Palermo, Via Archirafi 28, 90123 Palermo, Italy; 4 Department of Developmental and Molecular Biology, Albert Einstein College of Medicine, 1300 Morris Park Ave, Bronx, NY 10461, USA; fabrizio.biundo@einsteinmed.edu

Due to the increase in life expectancy, the aging population around the globe has been growing significantly and is estimated to triple by 2050. Cellular senescence has been indicated as one of the molecular processes involved in age-related diseases, including cardiovascular diseases, cancer, neurodegenerative diseases and diabetes, which have become a primary health issue challenging the health care system worldwide. During the last decades, internal and external causes of senescence have been characterized, such as organelle deterioration and the senescence-associated secretory phenotype (SASP), a cellular phenotype characterized by an increased expression and secretion of proinflammatory cytokines and chemokines. Moreover, oxidative stress has been shown to play an important role in exacerbating senescence by promoting the accumulation of reactive oxygen and nitrogen species and by disrupting protein turnover.

The majority of anti-aging research has been investigating metabolic pathways associated with senescence to define therapeutic strategies to slow the progressive decline of age-related pathologies. Among these strategies, a balanced diet including antioxidant phytochemicals and a healthy lifestyle have also been described to have beneficial effects in fighting aging and its related disorders.

The present editorial summarizes the most recent findings published in *Cells* that analyze cellular senescence in different cell and physiological models.

The extensive DNA damage induced by ionizing radiation (IR) is responsible for prolonged DNA damage response and premature senescence. To investigate the mechanism underlying the radiation-induced DNA damage and senescence, and in particular, the connections between altered chromatin compaction, nuclear envelope destabilization and nucleo-cytoplasmic chromatin blebbing, Freyter and colleagues combined mass-spectrometry-based proteomics with high-resolution imaging microscopy. The authors determined that the senescent phenotype observed was associated with disruption of nuclear lamin, which leads to extensive chromatin remodeling and consequent destabilization of the nuclear membrane with release of chromatin fragments into the cytosol. The morphology of a senescent fibroblast was further characterized by block-face scanning electron microscopy (SBF-SEM). This method allowed the high-resolution, 3-dimensional (3D) reconstruction of the complex nuclear shape and located the segregation of nuclear blebs fusing with lysosomes. Interestingly, the authors identified nanotubular channels formed in lamin-perturbed nuclei of senescent fibroblasts that could play a potential role in senescence [1].

Monoamine oxidase (MAO) plays a major role in neuroinflammation, and therapies targeting MAO could represent a new frontier for neurodegenerative diseases. The small-molecule drug tranylcypromine, an inhibitor of MAO, is currently used as an antidepressant and in the treatment of cancer. Park and colleagues detected that tranylcypromine plays a preferential role in altering lipopolysaccharide (LPS)-induced proinflammatory cytokine levels in BV2 microglial cells, but not in primary astrocytes. Mechanistically, tranylcypromine modulated LPS-mediated TLR4/ERK/STAT3 signaling. These data were confirmed in vivo. Tranylcypromine significantly reduced microglial activation and proinflammatory cytokine levels in wild-type mice treated with LPS. In the Alzheimer mouse model 5xFAD, this molecule significantly decreased microglial activation but had limited effects on astrocyte activation. In summary, this study indicates tranylcypromine as a potential therapeutic that can suppress LPS- and Aβ-induced neuroinflammatory responses both in vitro and in vivo [2].

Endothelial senescence is a risk factor in age associated cardiovascular disease. In their study, Jung and colleagues examined the combinatory effects of Rho-associated kinase (ROCK) inhibitor Y-27632 and mesenchymal stem cell-derived conditioned medium (MSC-CM) on the proliferation and senescence of rabbit corneal endothelial cells (rCECs). rCECs cultured in the presence of the inhibitor and MSC-CM showed a higher passage number, cell proliferation, and low senescence. rCECs on collagen type I film showed high expression of tight junction. This combined treatment significantly reduced senescence markers including β-galactosidase, LMNB1 and MAP2K6 expression. In contrast, cell cycle checkpoint genes such as CDC25C, CDCA2 and CIP2A did not vary in the combinatory medium but were significantly downregulated in either the ROCK inhibitor or MSC-CM alone. These results demonstrated that the synergistic effect of Y-27632 and MSC-CM on corneal endothelial cell proliferation and senescence could represent a significative potential intervention for the regeneration of human corneal endothelial tissue [3].

The vascular endothelium is involved in the control of the arterial structure and vasodilatory, thrombolytic, and vasoprotective functions. Its dysfunction is associated with risk factors for the development of several human vascular diseases, such as atherosclerosis, hypertension and stroke, peripheral arterial disease, metabolic syndrome and diabetes. Pin1 has been reported to directly control NO production by interacting with endothelial nitric oxide synthase (eNOS) in a phosphorylation-dependent manner, regulating its catalytic activity as well as regulating other intracellular players, such as VEGF and TGF-β, thereby impinging upon NO release. Since the molecular mechanisms through which Pin1 may derange vascular homeostasis remain largely unknown, Fagiani and collaborators applied a model-based network approach based on data derived from humans to recapitulate the mechanistic connection between Pin1 and the biological pathways involved in vascular processes. Pin1 emerges as a plausible critical driver of vascular cell proliferation, apoptosis and inflammation, with an implication for many vascular diseases (e.g., diabetes, atherosclerosis, hypertension and cardiac hypertrophy). Therefore, understanding the role of Pin1 in vascular homeostasis seems to be important in terms of finding new possible therapeutic players and targets, including those affecting the elderly (such as small and large vessel diseases and vascular dementia) or those promoting the full expression of neurodegenerative dementing diseases [4].

Hypertension, one of the most common comorbidities causing chronic endothelial dysfunction and disruption of the integrity of endothelial cells (ECs) and contributing to oxidative stress, inflammation and atherosclerosis, has been recognized as a leading risk factor for cognitive decline. The beneficial effect of metformin, an oral antidiabetic drug, on high-glucose-induced cell death, cell permeability and generation of reactive oxygen species in human brain microvascular endothelial cells (ECs) and on cognitive impairment in frail hypertensive patients, has been investigated by Mone and colleagues. The authors found that hyperglycemic patients treated with metformin had a significantly better Montreal Cognitive Assessment (MoCA) score than hyperglycemic patients treated with insulin. Interestingly, metformin exerted beneficial effects on human brain microvascular ECs [5]. In a recent study, Bima et al. investigated the potential protective role of vitamin D supplementation in obese albino rats, suggesting an interplay between vitamin D deficiency and increased cellular senescence in the pathogenesis of obesity-associated subclinical atherosclerosis [6].

Further insights of the role of senescence in atherosclerosis were discussed by Machado-Oliveira and colleagues in a comprehensive review. The authors analyzed key senescence-related alterations of the major intracellular organelles and highlighted the role of relevant cell types for senescence and atherogenesis, providing an updated analysis of therapeutic approaches including clinically relevant experiments using senolytic drugs to counteract atherosclerosis [7].

A marked senescent phenotype has been reported in renal, prostate and bladder disorders. The unwanted effects of persistent senescent cells have been partially linked to a senescence-associated secretory phenotype (SASP), a status characterized by the release of a variety of factors causing chronic inflammation, extracellular matrix remodeling and fibrosis. In this review, the authors summarized the molecular mechanisms of senescence and their implications in renal and urinary tract conditions, and analyzed the differential effects of transient versus persistent cellular senescence. They also suggested and listed potential therapeutics targeting senescence in these diseases [8].

Cellular senescence is a stress-induced state of irreversible cell cycle arrest accompanied by organ dysfunction that has become an increasingly critical aspect in aging research. Since the understanding of differences in genetic background in the development of cellular senescence is lacking, Liao and collaborators provided new information using primary tubular epithelial cells (PTECs) derived from genetically different inbred mouse strains. Kidneys from 129S1, B6, NOD, NZO, CAST and WSB mice were used to isolate PTECs, which were monitored for expression of typical senescence markers at 3 and 10 days after pro-senescent gamma irradiation. These different markers were analyzed individually and in combination to generate a senescent signature score for each mouse strain. Data highlighted that the rate of stress-promoted senescence induction in cultured primary kidney cells is different in five mouse strains, indicating an importance of the underlying genetic background. The highest senescence values for PTECs were found in WSB mice, whereas the lowest ones were from 129S1 mice. These results not only provide new insights on the genetic diversity and on the development of senescence but can also explain heterogeneity in existing data. Therefore, they should be considered when designing new experiments and as the basis for further investigation to identify candidate loci driving pro- or anti-senescent pathways [9].

The progressive accumulation of apoptosis-resistant and secretory active senescent cells (SCs) in animal- and human-aged tissues has been linked to age-related diseases including cancer, neurodegenerative disorders, and metabolic syndromes. Recently, thanks to their unique characteristics, nanomaterials have been indicated as innovative tools in theranostics. Despite this, the role of this technology in modulating cell senescence remains elusive.

To bring more clarity to this field, Adamczyk-Grochala and Lewinska analyzed this innovative nanotechnology-based strategy in relation to the prevention of age-related conditions. The delivery of therapeutic compounds capable of preferentially killing SCs (nano-senolytics) and/or modulating a proinflammatory secretome (nano-senomorphics/nano-senostatics) combined with recent examples of SC-targeted nanomaterials and the mechanisms underlying different aspects of the nanomaterial-mediated senolysis was discussed by the authors [10].

Age-related hearing loss (ARHL) is among the most common disorders affecting older people and is characterized by a progressive form of bilateral hearing loss. Although the mechanisms causing ARHL remain mostly unclear, the cross-link between hearing loss and endoplasmic reticulum (ER) stress has been recently demonstrated by in vitro and in vivo experiments, and the critical role of heat shock factor 1 (HSF1) has also been suggested. Indeed, HSF1 responds to ER stress by controlling the refolding or degradation of misfolded or unfolded proteins, respectively, by increasing heat shock protein (HSP) levels such as Hspa1a (HSP70) and Dnajb1 (HSP40). Lee and collaborators showed in premature senescence, and in heat shock-exposed cell models and ARHL cochlear tissues, there were increased levels of the ER stress marker proteins (p-eIF2 α and CHOP) and reduced levels of HSF1 and various HSPs including HSP70 and HSP40, which were significantly downregulated, thus indicating that HSP70 and HSP40 were the main targets of HSF1 and that these co-chaperones play an important role in reducing ER stress. Moreover, HSF1 overexpression showed significant hearing protection effects, further supporting that HSF1 expression may be a main cause of age-related hearing loss and HSF1 can function as a mediator to prevent ARHL by decreasing ER stress-dependent apoptosis in the aging cochlea [11].

Sex steroids and corticosteroids can regulate different physiological functions through their interaction with specific nuclear receptors and, consequently, interference with hormone activities (e.g., the exposition to exogenous hormone-active substances such as endocrine-disrupting chemicals (EDCs) or deregulation of their production and downstream pathways) can affect the regulation of their correlated pathways and trigger the neoplastic transformation and hormone-sensitive cancer development. Although nuclear receptors account for most hormone-related biologic effects, membrane receptors are emerging for their ability to mediate steroid hormone effects and to be involved in the development of hormone-sensitive cancers. Molecular mediators of the androgenic action include different plasma membrane receptors, namely, the zinc transporter member 9 (ZIP9), the oxoeicosanoid receptor 1 (OXER1), the G protein-coupled receptor family C group 6 member A (GPRC6A), the Ca2+ channel transient receptor potential cation channel subfamily M (melastatin) member 8 (TRPM8) and the L-type voltage-dependent calcium channel (CaV1.2). Molecular mediators of estrogenic action are located at the cellular plasma membrane and include the G protein estrogen receptor (GPER), ERx, ER-X and Gq-coupled membrane estrogen receptor (GqmER). In addition, voltage-gated sodium channel Nav1.2 is also reported to bind estrogens and to activate molecular cascades upon their binding. Membrane progesterone receptors that mediate nontraditional progesterone actions are classified into two groups: the class II progestin and adipoQ receptor (PAQR) family (also called membrane progesterone receptors, mPRs) and the b5-like haem/steroid-binding protein family (also called membrane-associated progesterone receptors, MAPRs). To elucidate the role of these membrane receptors as key players in shaping the cancer environment and, therefore, as potential drug targets, Masi and collaborators showed the membrane receptors’ pivotal role in mediating the effects of steroid hormones and hormone-active substances in both physiologic and pathologic contexts by collecting pre-clinical and clinical data; thus, providing the first critical and in-depth view in the context of hormone-sensitive cancers [12].

The possible interconnection between the eye and the central nervous system (CNS) has been a topic of discussion for several years, although the existence of a strong link is supported by evidence that ocular alterations existing in various neurodegenerative pathologies of the CNS and visual manifestations sometimes precede central symptoms. Although both organs can be affected by neurodegeneration, it mainly occurs in the form of cognitive diseases in the brain, such as Alzheimer’s or Parkinson’s diseases, whereas it occurs in the form of glaucoma in the eyes. Moreover, neurodegeneration and cell loss associated with both CNS and retina disorders involve several common etiological factors such as oxidative stress, neuroinflammation, proteolytic degradation, dysregulation of ocular hemodynamic parameters, trans-synaptic degenerative changes, genetic causes and aberrant cellular signaling. Early signs of retinal damage are present in Parkinson’s and Alzheimer’s diseases, as well as in multiple sclerosis. Moreover, age-related macular degeneration (AMD) and AD share the same biomarkers, thus suggesting that having biomarkers available in the preclinical phase, to signal the brain pathological process in progress, may be essential to obtain effective therapies, and even more if the biomarkers are sensitive to therapeutic treatments. Since the prevalence of neurological diseases increases dramatically with age and could negatively impact people’s quality of life, Marchesi and collaborators provided evidence of the interconnection between retina and cortical areas to understand the mechanisms of the onset of neurologic diseases. They also deeply discussed the characteristics of the ocular illnesses, focusing on the relationship between the eye and the brain to provide a better comprehension that could help in future new therapies, thus reducing or avoiding loss of vision and improving quality of life [13].

Cellular senescence plays an essential role in age-related changes of the adrenal cortex, as the circulating levels of aldosterone and adrenal androgen gradually descend, whereas those of cortisol increase with aging; however, the detailed mechanisms are still unknown. The recent advances related to cellular senescence in adrenocortical biology and its associated disorders were summarized by Gao et al. [14].
